# Electronic Immunization Registry in Rwanda: Qualitative Study of Health Worker Experiences

**DOI:** 10.2196/53071

**Published:** 2024-05-28

**Authors:** Thaoussi Uwera, Mahima Venkateswaran, Kiran Bhutada, Eleni Papadopoulou, Enock Rukundo, David K Tumusiime, J Frederik Frøen

**Affiliations:** 1 Centre of Excellence in Biomedical Engineering and eHealth University of Rwanda Kigali Rwanda; 2 Centre for Intervention Science for Maternal and Child Health (CISMAC) University of Bergen Bergen Norway; 3 Global Health Center Albert Einstein College of Medicine Bronx, NY United States; 4 Global Health Cluster, Division for Health Services Norwegian Institute of Public Health Oslo Norway

**Keywords:** childhood immunization, electronic immunization registry, digital health interventions

## Abstract

**Background:**

Monitoring childhood immunization programs is essential for health systems. Despite the introduction of an electronic immunization registry called e-Tracker in Rwanda, challenges such as lacking population denominators persist, leading to implausible reports of coverage rates of more than 100%.

**Objective:**

This study aimed to assess the extent to which the immunization e-Tracker responds to stakeholders’ needs and identify key areas for improvement.

**Methods:**

In-depth interviews were conducted with all levels of e-Tracker users including immunization nurses, data managers, and supervisors from health facilities in 5 districts of Rwanda. We used an interview guide based on the constructs of the Human, Organization, and Technology–Fit (HOT-Fit) framework, and we analyzed and summarized our findings using the framework.

**Results:**

Immunization nurses reported using the e-Tracker as a secondary data entry tool in addition to paper-based forms, which resulted in considerable dissatisfaction among nurses. While users acknowledged the potential of a digital tool compared to paper-based systems, they also reported the need for improvement of functionalities to support their work, such as digital client appointment lists, lists of defaulters, search and register functions, automated monthly reports, and linkages to birth notifications and the national identity system.

**Conclusions:**

Reducing dual documentation for users can improve e-Tracker use and user satisfaction. Our findings can help identify additional digital health interventions to support and strengthen the health information system for the immunization program.

## Introduction

In 2021, a reported 18.2 million infants worldwide did not receive basic immunization, and an additional 6.8 million were only partially vaccinated, with associated higher deaths in low- and middle-income countries (LMICs) [1,2]. Health systems worldwide are adopting digital tools to improve immunization service provision as well as monitoring [3]. Digital health interventions (DHIs) have the potential to improve the management and use of health information to enhance health worker performance and provision of care and ultimately improve health outcomes [4,5]. In LMICs, electronic immunization registries (EIRs) were initiated to support improved vaccination coverage among children, primarily through better tracking of children by combining vaccine information from different sources into a single digital record [6,7].

DHIs (in the form of EIRs) are important for immunization programs. For clients, they can help to remind families through SMS text messaging when immunization is due or has been missed. For health workers, they can help ensure that children get the vaccinations they need, improve and simplify the reporting of immunization data, identify high-risk populations for targeted interventions, and allocate resources efficiently and effectively [5-7]. EIRs, enhanced by data-driven DHIs, can help the immunization program achieve its goals of effective immunization coverage and real-time data for decision-making. EIRs can serve their purpose for immunization programs even better if integrated and synergized with DHIs for other programs such as Civil Registration and Vital Statistics (CRVS) and the national identification system. For instance, registration of all newborn babies in EIRs can improve tracking of immunization status and monitoring coverage [7]. EIRs integrated with other programs can strengthen other health services for children by providing a database of newborn babies in the population. Examples include newborn metabolic screening and childhood nutrition programs for the identification and referral of malnourished children [7].

Despite the many opportunities, several challenges hinder the effectiveness of EIRs in LMICs, such as the increased burden of data collection for health workers, which is the result of maintaining paper and digital documentation and reporting systems [7]. The implementation of EIRs, similar to all DHIs, should be aligned with the needs, both in terms of addressing the concerns of the intended users and being relevant to the users [8]. However, there is limited evidence on how to implement digital tools most effectively and sustainably across the full range of health systems [9]. The World Health Organization has highlighted the need for implementation research to identify the crucial factors that affect the implementation of DHIs for health system strengthening [5]. Implementation research can provide a systematic understanding of users’ perceptions and experiences and thus enhance the usability and acceptability of DHIs.

In Rwanda, children from 0 to 15 months of age are provided with vaccines against 11 infections according to the Expanded Program on Immunization (EPI), namely, tuberculosis, poliomyelitis, diphtheria, tetanus, measles, pertussis, hepatitis B, *Haemophilus influenzae* type B, rubella, *Streptococcus pneumoniae*, and rotavirus [10]. The latest report from the Global Alliance for Vaccines and Immunization from 2017 identified issues with the immunization health information system such as data quality, population denominators based on projections from census data, and implausible coverage rates of more than 100% [11], similar to other contexts in eastern and southern Africa [12]. Incidents of vaccine dropouts and incomplete immunization, particularly for Pentavalent 3, were also identified. Significant geographic variations in immunization rates were reported, with 1 district in the northern part of the country reporting an overall coverage rate of as low as 88% [11,13].

The introduction of an EIR, known as e-Tracker, was launched in 2019 with the goal of improving overall data quality, data availability for monitoring of immunization defaulters or dropouts, and ultimately increasing immunization coverage [14]. The newly implemented e-Tracker has not yet been subject to research-based evaluations. The aim of this study was to assess the extent to which the immunization e-Tracker responds to stakeholders’ needs and identify key areas for improvement in Rwanda’s childhood immunization program.

## Methods

### Study Setting

This study was conducted among immunization nurses and data managers. Supervisors were included at the district hospital level. Health facilities were randomly selected from 5 districts in Rwanda—Gasabo, Rwamagana, Kamonyi, Gicumbi, and Rubavu, 1 from each of the 4 provinces and the City of Kigali of Rwanda. Gicumbi district, which is in the north of Rwanda, has 16 health centers; Kamonyi, in the south, has 13 centers; Rwamagana, in the east, has 15 health centers; Rubavu, in the west, has 13 centers; and Gasabo, in the central city of Kigali, has 16 health centers. In Kamonyi district and Gicumbi district, the routine immunization coverage rates for Pentavalent 3 and measles-rubella 1 in 2018 were 84% and 85%, respectively, while the coverage rate was higher than 89% in the remaining 3 districts. Gicumbi, Gasabo, and Kamonyi were among the districts with the largest percentage of underimmunized children, especially for the third dose of Pentavalent. The e-Tracker was introduced and operationalized in health centers in all districts of Rwanda in 2019.

The study participants were primary users of the immunization e-Tracker, either entering data or using the data: immunization nurses, data managers, and EPI supervisors.

Immunization-related services are organized at different levels of the health system. At the village level, community health workers engage with residents to raise awareness about childhood immunization. All primary health care services, including childhood immunization, are decentralized to the health center level. Immunization is provided at the health centers by immunization nurses or at health posts by the same nurses through community outreach in hard-to-reach areas. There are 499 health centers and 476 health posts in Rwanda [15]. More than 90% of children are immunized at the health center. All immunization sites (centers and posts) have weekly schedules of immunization days.

A health center typically has 2 immunization health workers, a nurse in charge of immunizations, and an assistant to deliver vaccines and keep records of all information pertaining to immunizations.

**Figure 1:**
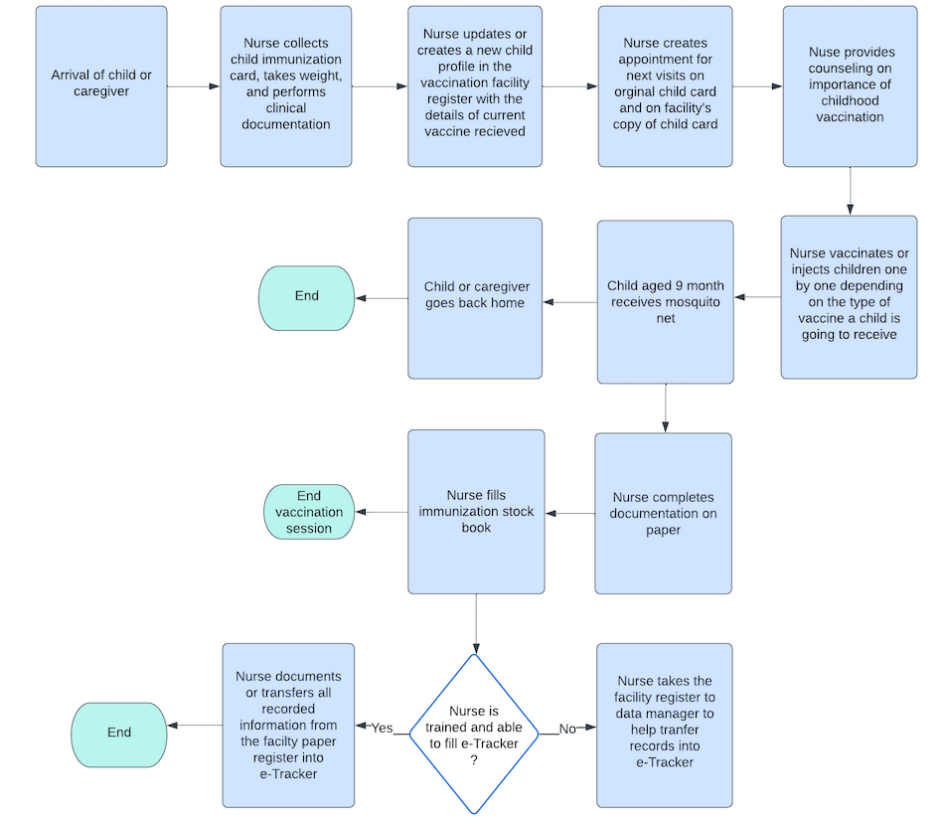
Workflow of immunization pertaining all the duties of a health worker (immunization nurse) on a vaccination day.

**Figure 2:**
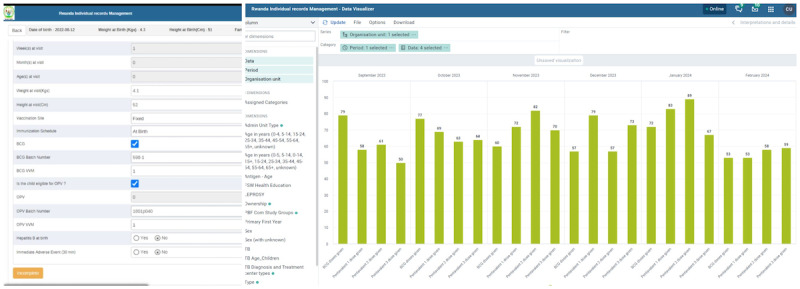
Immunization e-Tracker showing the client registration page and data visualization dashboard.

Figures 1 and 2 show the workflow of immunization at the health facility and the e-Tracker registration process and data visualization, respectively.

### e-Tracker Implementation and Use

Implementation of the e-Tracker started in 2019 and was operational in all public health facilities. The e-Tracker runs on the District Health Information Software 2 (University of Oslo) platform, one of the most widely used digital health information systems globally [16]. Three cadres of health workers were trained to use the e-Tracker—immunization nurses, data managers, and EPI supervisors. All individual information are first recorded on 2 sets of paper-based forms: the child’s immunization cards and the health center’s immunization paper registers. The immunization nurse or the data manager then transfers the same information from the paper registers to the e-Tracker. At the end of the month, a set of predefined data is aggregated onto paper reporting forms by immunization nurses and handed over to the data manager, who then enters these data into the aggregate reporting system built in a separate instance of District Health Information Software 2, as a part of the health management information system (HMIS).

EPI supervisors, located at the district hospitals, use the e-Tracker to assess the progress of health facilities by comparing the number of children registered as successfully vaccinated on each indicator against the monthly target provided to the health center (Table 1). The target is an estimate population based on the expected number of births in the area based on census data.

Users who have technical issues with e-Tracker can contact the central help desk. Phone calls or WhatsApp groups are typically used to resolve simple technical issues such as password reset, and for complicated issues, through visits to health centers. Immunization nurses from the health centers have a joint WhatsApp group with their respective EPI supervisors where they communicate issues regarding immunization and e-Tracker–related technical support in their district.

**Table 1:** Intended use and user roles in the immunization e-Tracker.

User	Intended use and user roles in the e-Tracker
Immunization nurse^a^	Data entry and registration of new children for immunization Update and follow up on subsequent immunizations until a child has completed his or her vaccination calendar
Data manager^a^	Data entry and registration of new children for immunization Update and follow up on subsequent immunizations until a child has completed his or her vaccination calendar Generate reports of comparisons of the health center’s immunization coverage rate against the target
EPI^b^ supervisor	Review reports from all health centers in the district catchment area and provide recommendations and feedback for improvement based on the data

^a^Data entry tasks could be shared by immunization nurses and data managers.

^b^EPI: Expanded Program on Immunization.

### Study Design and Sampling

This study is an implementation research design that used descriptive qualitative methods [17,18] and formative evaluation to assess the extent to which the immunization e-Tracker responds to stakeholders’ needs and identify key areas for improvement [19]. This was done through key informant interviews. The Human, Organization, and Technology–Fit (HOT-Fit) evaluation framework guided the data collection and analysis [20]. We chose the HOT-Fit framework because it has the potential to evaluate health information systems; encompasses comprehensive dimensions; and measures the fit between technological, human, and organizational aspects, all of which are critical for system adoption [20].

To select a sample of districts, we first assessed data reports retrieved from the e-Tracker and the national HMIS in the first 3 months of 2020 for all 30 districts in Rwanda. Four immunization indicators—Bacille Calmette-Guérin (BCG) and Pentavalent (penta) first, second, and third doses (penta1, penta2, and penta3)—were reviewed by a program manager together with a researcher (TU) to calculate completeness of data in the e-Tracker (e-Tracker–reported indicator and HMIS-reported indicator). We then selected 5 districts as follows: 1 district among the best performers (Rwamagana district >80%), 1 from the worst performers (Rubavu district <15%), and 3 districts that were in the middle (Gasabo, Gicumbi, and Kamonyi districts: 50%-60%). We randomly included 6 health centers from each of the 5 districts. From this pool of health centers, key informants and participants were purposively sampled among primary users of the e-Tracker. To cover the variation of sites across the districts appropriately, we recruited 1 nurse and 1 data manager from 1 district before moving to the next to diversify the data collected.

### Data Collection

This study was carried out in accordance with COREQ (Consolidated Criteria for Reporting Qualitative Research) guidelines (Multimedia Appendix 1) [21].

Based on the 3 constructs of the HOT-Fit framework, we created study-specific definitions for each of the constructs (Table 2) and formulated an interview guide with open-ended questions (Multimedia Appendix 2). Three pilot key informant interviews were conducted with immunization nurses and data managers in 1 district (Gasabo) to validate the tool prior to data collection. We further refined the questions in the guide based on the findings from these interviews (Multimedia Appendix 2).

**Table 2:** Specific domains of evaluation of the e-Tracker based on the constructs of the HOT-Fit^a^ framework.

HOT-Fit constructs and definitions	Study constructs and definitions
Technology: Meets the need of the projected users, is convenient and easy to use, and fits the work patterns of the professionals for whom it is intended and the overall health system	System quality Associated with system performance: ease of use, ease of learning, response time, usefulness, system flexibility, and security Information quality User perspectives and quantitative data: completeness, availability, accuracy, reliability, timeliness, relevance, and consistency Service quality Service delivered: technical support, quick responsiveness, assurance, empathy, and follow-up service
Human: The person who uses and the use of information output such as reports	System use Concerned with the frequency and breadth of health information system inquiries and functions: system users, their levels of use, training, knowledge, belief, expectation, acceptance, or resistance User satisfaction Evaluation of users’ experience in using the system and the potential impact of the system: perceived usefulness, enjoyment, overall satisfaction and satisfaction with specific functions, and decision-making satisfaction
Organization: Nature and factors of a health care institution	Structure Nature (type and size), management and communication, clinical process, and workflow process. Leadership, top management support, etc Environment Financial source, government, politics, and type of population being served
Net benefits	Quality of care, clinical impact, impact on patient care and communication, and facilitation of information access

^a^HOT-Fit: Human, Organization, and Technology–Fit.

Separate interview guides were used for each category of participants. The interview questions were formulated based on each user’s role both in the immunization program and the e-Tracker system. For instance, we asked questions related to user-specific employment and how e-Tracker is related to his or her job. Some e-Tracker technical questions were similar such as whether e-Tracker was easy to use, easy to learn, or about how e-Tracker responds (response time). One author (TU), a current PhD candidate, with experience in IT conducted 14 in-person, in-depth interviews with key informants (e-Tracker end users in primary health care centers and EPI supervisors in their affiliated district hospitals). Interviews were conducted with only the key informant and the interviewer present. The interviews were conducted in Kinyarwanda, took place over approximately 1 hour, and were audio recorded. No notes were taken. The audio was then transcribed in Kinyarwanda and translated into English by a bilingual professional. A group of 2 researchers (TU and ER) reviewed the translations for accuracy. The study team met on a weekly basis to evaluate the data collection process. After 4 interviews per key informant category, the data collector began hearing information repetition. The research team advised undertaking 1 more interview per participant category to ensure that no new findings were discovered. Data saturation was confirmed, and data collection was stopped. No repeat interviews were carried out.

### Data Analysis

Translated interview transcripts were uploaded into NVivo 12 (Lumivero). Based on the HOT-Fit framework, a codebook was developed by the team through discussion. Using this agreed-upon codebook, 2 researchers (TU and ER) individually coded the data. A deductive coding style was applied to our data. Discrepancies in coding were discussed and resolved by the team.

### The HOT-Fit Framework

After coding was completed by both researchers, the team compiled the relevant data extracts. We performed a framework analysis and worked together to place the extracted data within the HOT-Fit framework [20,22]. We analyzed interview transcripts to find all possible codes from all participants. We identified and summarized codes in accordance with constructs of the HOT-Fit framework and study-specific domains (Table 2). NVivo 12 analysis software was used to manage themes and codes.

### Author Reflexivity

Prior to data collection, the interviewer and research team had minimal contact with participants (stakeholder engagement session). The participants were informed that the purpose of the study was to gather their views and experiences on e-Tracker use to assess how the immunization e-Tracker responds to stakeholders’ needs and identifies areas for improvement. They were also informed that this was part of a larger project studying the design and implementation of DHIs to improve childhood immunization in Rwanda. Authors entered this study with the belief that an e-Tracker had the potential to positively impact care providers’ experiences; however, it took effort to prevent personal bias during data analysis. Due to COVID-19 restrictions, member checking was completed with 1 key informant from each category.

### Ethical Considerations

This study was approved by the Rwanda National Ethics Committee (1011/RNEC/2020), the Norwegian (West) Regional Committee for Medical and Health Research Ethics (251925), and the Rwanda Ministry of Health’s National Health Research Committee (reference NHRC/2021/PROT/002). All methods were performed in accordance with relevant guidelines and regulations by the World Medical Association Declaration of Helsinki—ethical principles for medical research involving human subjects [23].

The participants were informed about the study objectives, their voluntary participation, and their right to refuse participation at any time. The written informed consent form was obtained from each participant after getting an explanation about the research purpose and confirming their participation in the study. The interviews took place in a safe room with the office door locked at the health facility. The recorded information was transcribed and anonymized. The audio recording device could only be accessed via a security code by the lead author (TU).

## Results

### Overview

In total, 14 e-Tracker users were interviewed (Table 3), including 5 immunization nurses, 5 data managers, and 4 EPI supervisors (1 EPI supervisor declined being interviewed due to clinical COVID-19 work). Most of the immunization nurses were female (4/5, 80%) and had more than 10 years of work experience (3/5, 60%). In contrast, data managers were mostly male (4/5, 80%), younger, and had work experience of 5 years or less (4/5, 80%). Half of the supervisors (2/4, 50%) were female. The supervisors had varying levels of work experience (Table 3). We present our findings based on the constructs of the HOT-Fit framework.

**Table 3:** Characteristics of study participants.

Characteristics		Immunization nurses (n=5)	Data managers (n=5)	Supervisors (n=4)
**Sex, n (%)**				
	Male	1 (20)	4 (80)	2 (50)
	Female	4 (80)	1 (20)	2 (50)
**Age range (years), n (%)**				
	25-35	1 (20)	2 (40)	1 (25)
	36-45	2 (40)	3 (60)	0 (0)
	46-55	2 (40)	0 (0)	2 (50)
	56 and older	0 (0)	0 (0)	1 (25)
Age (years), mean (SD)		43 (8.29)	35 (5.89)	50 (10.23)
**Field of study, n (%)**				
	Nursing	5 (100)	2 (40)	0 (0)
	Laboratory	0 (0)	1 (20)	0 (0)
	Computer science	0 (0)	1 (20)	0 (0)
	Public health	0 (0)	1 (20)	3 (75)
	Midwifery	0 (0)	0 (0)	1 (25)
**Working experience (years), n (%)**				
	≤5	1 (20)	4 (80)	2 (50)
	6-10	1 (20)	1 (20)	1 (25)
	>10	3 (60)	0 (0)	1 (25)

### Technology

#### System Quality

Data managers and supervisors reported that the e-Tracker was not a complex system. Two (40%) of 5 nurses perceived the e-Tracker as complex due to limited skills of computer literacy (Table 4: section A, construct 1).

**Table 4:** Summary of main findings in accordance with the HOT-Fit framework and quotes from key informant interviews.

Construct number and main findings			User’s quotes
**Section A: Technology**			
	**System quality**		
	1. Ease of learning	“...that system [e-Tracker] is not difficult to use, except that it is not easy for everyone because there are some health centers for example that have immunization nurses who do not know how to use the computer.” (EPI^a^ supervisor 1)	
	2. Better data security than paper registers and forms	“e-Tracker is a secure system protected by personal credentials; it is not like paper registers where anyone can access.” (Data manager 5)	
	3. Missing technical functionalities	“...as a person who is in the field and using it [e-Tracker] frequently, I realize that there are some functionalities that the e-Tracker is lacking. For example, it does not show me the next appointment for someone’s vaccination or the list of who the nurses should be seeing today.” (Data manager 5)	
	4. Not compatible for community outreach	“...internet connection that is not available, lack of outreach support—all these are challenges with using the e-Tracker.” (Immunization nurse 1)	
	5. Connectivity issues and slow system response	“Things related to e-Tracker are slow, definitely slow. This is a challenge we usually face.” (Immunization nurse 2)	
	**Information quality**		
	6. Incomplete and unreliable data	“There are times when you register a child and when you go back to search him or her, you find that the actual information is not complete, or you find that the e-Tracker contains a duplicate of the child’s records.” (Immunization nurse 3)	
	7. Increased documentation workload	“I may fail to get time for instance, and they shift me to provide another health service, but, because there is much information that needs to be entered and I am responsible for that, I go quickly and take like one hour after work, or I come early in the morning to enter them.” (Immunization nurse 4)	
	**Service quality**		
	8. Delays in getting technical support	“It is difficult to get technical assistance because it is from central level and nowhere else...if the problem is simple like the system is off and then back on, those ones are quick and can be done on a phone call or WhatsApp, but bigger technical issues take time.” (Data manager 2)	
	9. Alternative communication lines	“...talking about the other [communication] chain...I just call my superior at the hospital, and he conveys it to the central level technical team...and they gradually communicate with each other, and the information reaches us.” (Data manager 4)	
**Section B: Human**			
	**System use**		
	10. Does not meet the intended purpose	“What I expected from e-Tracker up to now, I can say that I have not yet seen its results. This may be due to other challenges, but the functionalities required by the nurses to use the e-Tracker well and properly are not yet available.” (Supervisor 1)	
	11. Suboptimal use due to increased documentation workload	“This e-Tracker system is expected to be used by immunization nurses; it has apparently increased their work, which was not easy. That is simply to say, this is beyond their capacity.” (Supervisor 1)	
	**User satisfaction**		
	12. General dissatisfaction with the e-Tracker	“...Discriminating children's cards increases job, in e-Tracker it is simple; just search child and find him easily, but the use of e-Tracker did not stop papers, you complete all existing paper books and forms and then go complete e-Tracker.” (Immunization nurse 2)	
**Section C: Organization**			
	**Structure**		
	13. Lack of effective training processes	“...data manager who received training has gone, the one who replaced him does not actually know to use e-Tracker, he often called me asking, ‘where can I click on?’...you realize that it is slowly by slowly.” (Immunization nurse 2)	
	14. Lack of support for health workers in using technology	“...it happens that you register a child and when you go back to search for him [in the e-Tracker], you miss him simply because you do not know if it is a connection problem, or a low knowledge regarding how to search for him.” (Immunization nurse 1)	
	**Environment**		
	15. Performance-based financing	“We have many duties, and there are so many systems at health center...they come and say we give you PBF after seeing in the system how many children you have entered, and it is understandable that you will not receive any money if you didn’t register any child.” (Immunization nurse 3)	
**Section D: Net benefits**			
	16. Perceived current benefits	“...the e-Tracker has a dashboard for data analysis. Like now, I sometimes say, let me see how many children we have registered this month; for the first, the second and the third dose of Penta, for instance.” (Data manager 3)	
	17. Perceived future benefits for HWs^b^	“e-Tracker has made nothing easier for me. Instead, it has complicated things. Perhaps there is value in the e-Tracker if all these papers and books are removed. Then you may find that the e-Tracker will bring benefits.” (Immunization nurse 3)	

^a^EPI: Expanded Program on Immunization.

^b^HW: health worker.

Data security in the e-Tracker was generally perceived as satisfactory and better than data security using paper registers (Table 4: section A, construct 2). However, users reported several shortcomings. They cited the lack of several technical functionalities such as client lists, lists of defaulters, unspecific search and register functions, automated routine reports, and linkage to other systems such as birth notification and the national identity system (Table 4: section A, construct 3). Users expressed the need for a more flexible data entry tool that can operate offline, such as handheld tablets instead of desktop computers, to use during community outreach. They also cited poor connectivity and solely relying on health center–purchased internet as one of the most important reasons for the suboptimal use of the e-Tracker (Table 4: section A, construct 4). For system response time, 4 (80%) of 5 health workers and 4 (80%) of 5 data managers reported that the e-Tracker responds slowly. The remaining interviewees, particularly supervisors, located at hospitals with better internet connectivity, reported the opposite that the e-Tracker had a quick response time. Adequate support for network connectivity was lacking (Table 4: section A, construct 5). For example, immunization health workers at health centers were given modems, but they claimed that they were not given financial assistance for continued internet subscriptions.

#### Information Quality

Data in the e-Tracker were considered incomplete and unreliable and were not actively used by the immunization nurses (Table 4: section A, construct 6). Several underlying issues were identified as contributors to poor information quality. Users were required to document in the e-Tracker in addition to existing paper forms, which created double work. The double entry of data, combined with a mismatch between the data elements in the paper forms and the e-Tracker, results in users skipping some data fields in the e-Tracker. A common response with all users was the lack of time to complete documentations in the e-Tracker due to heavy workloads (Table 4: section A, construct 7). Two (40%) of the 5 interviewed immunization nurses were not trained in e-Tracker use, but even those who were trained and able to use the e-Tracker reported that the time allocated to them to fill the e-Tracker was insufficient. Three (60%) of 5 immunization nurses reported having to work overtime to enter data in the e-Tracker, 1 hour before or after work*—*a practice that users believed adversely affected data quality.

#### Service Quality

All interviewed nurses and data managers reported some form of delay in getting technical support (Table 4: section A, construct 8). Users’ responses on this issue suggest that they might prefer reporting issues to their supervisor, who could then facilitate communication with the central support team.

### Human

#### System Use

According to all interviewees, the e-Tracker did not meet overall user expectations. Further exploration revealed that users want a system that generates automated monthly reports and reduces documentation workload. The e-Tracker does not automatically generate any reports, and double documentation was identified as an important problem that impacted effective e-Tracker use (Table 4: section B, constructs 10, 11).

#### User Satisfaction

When asked whether they were satisfied with the e-Tracker, only 2 (40%) of 5 data managers said yes. The lack of technical functionalities and increased documentation workload were the leading causes of dissatisfaction for the data managers and health workers, respectively (Table 4 section B, construct 12).

### Organization

#### Structure

Users described quarterly data quality assessment workshops to encourage e-Tracker use by health workers. Such assessments are usually done by data managers, nurses, and their supervisors by reviewing paper reports and e-Tracker reports and comparing them to HMIS reports for selected vaccination indicators, such as BCG. Health workers reported not receiving enough support in navigating digital systems in general (Table 4 section C, construct 13) and highlighted the need for regular training sessions on how to use the e-Tracker and a plan to deal with staff turnover. The planned training for users in 2021 did not happen due to the COVID-19 pandemic.

#### Environment

Performance-based financing was provided to the health workers based on the number of newborn babies registered as BCG vaccinated in comparison with their reported number of BCG vaccinations. The interviewees alluded to this as a reason for entering data for this specific indicator into the e-Tracker rather than the indicators for other vaccines. Performance-based financing in this context is based on the number of children registered with BCG vaccination as a way of promoting the registration of newborn babies in the childhood immunization e-Tracker [24] (Table 4: section C, construct 15).

### Net Benefits

Participants acknowledged the potential benefits of an e-Tracker provided technical and implementation issues are addressed. For example, all EPI supervisors reported that the tool could be helpful to monitor children’s registration and vaccination status without visiting health centers physically. Two (40%) of 5 data managers reported using the e-Tracker for monitoring and evaluation in terms of vaccination coverage for their respective health centers. In contrast, all health workers did not report any net benefits from the current use, although they see that the e-Tracker may contribute positively to their work in the future (Table 4: section D, construct 17).

### Key Improvements

Textbox 1 provides a summary of the main recommendations for improvement of the e-Tracker based on our findings.

**Overall recommendations for key improvements highlighted by the users.**

**Immunization nurses**

- Better client search and register function
- Produce lists of expected and missed clients to avoid searching in paper registers
- Facilitate tracking a defaulter or a dropout child and remind parents of the missed appointments
- Improve connectivity
- Offline e-Tracker version that will make it easier to collect data in case of network outage, handheld devices to help immunization outreach in difficult-to-reach areas
- Regular training on e-Tracker use

**Data managers**

- Generate automatic monthly reports
- Link e-Tracker to other systems such as Civil Registration and Vital Statistics and national identification systems

**Expanded Program on Immunization supervisors**

- Additional trainings on analysis of e-Tracker data
- Offline e-Tracker version and more devices to support nurses’ work at primary health centers

## Discussion

### Principal Findings

This study explored stakeholders’ experiences and perceptions of using the e-Tracker for the Rwandan childhood immunization program. Users of the e-Tracker described several issues that hamper effective data entry as well as data use. Data in e-Tracker were reported to be incomplete and unreliable as result of dual documentation on paper and digitally.

Rwanda is one of the few countries in Africa to implement an EIR at scale. Implementation of the e-Tracker is a top priority for the childhood immunization program. Along with technological resources such as computers and modems, a top-level team and 3 cadres of trained health professionals from each health center across the nation are assigned to support the implementation indicating significant organizational support for change. EIRs allow for real-time monitoring of immunization status and provide data for decision-making, and their evaluations play a key role in identifying strategies to improve their use [25]. Our findings demonstrate the need for technical improvements to fit clinical practice and increase benefits, addressing implementation-related issues such as workflow matching, as well as training and user support. User-informed development of technical functionalities has been shown to be linked to higher adoption of health information systems in a systematic review of 55 studies [26]. Slow response times and delayed IT support adversely affected e-Tracker use in our study, factors also reported in other studies of digital information systems [27,28].

Creating an enabling environment for digital health systems by addressing issues such as training, and capacity strengthening in data entry and use, is equally important to ensure successful implementation [29]. Users cited a general dissatisfaction with the e-Tracker for several reasons including increased workload due to dual documentation and insufficient training. Several studies have reported similar dissatisfaction among users of digital health information systems in many cases as a result of the system’s inability to match existing work patterns [26]. On the other hand, users are typically more satisfied when information systems offer good quality data; the higher the quality of the data the higher the satisfaction [27,30]. Users in this study perceived the information in the e-Tracker to be inaccurate and incomplete in comparison with the paper records and registers. None of the entered digital information was used by data managers or nurses for clinical practice.

Immunization nurses are the intended users of the e-Tracker, although the current workflow involves secondary data entry in the e-Tracker by the data manager in several health centers. While data managers and supervisors stated some benefits of the e-Tracker for their work, immunization nurses reported no net benefits of the e-Tracker as it has been implemented in its current version. One of the reasons for this may be that the e-Tracker in its current form is not considered an essential part of the data ecosystem in the immunization health information system, particularly because the monthly reports are still paper based and not generated from the e-Tracker. In a setting such as Rwanda with scarce human resources for health, efficiency and costs are important considerations. Efficiency gains cannot be achieved unless health centers phase out paper immunization records and exclusively use the e-Tracker for data entry [31]. Similarly, a study conducted in Zambia and Tanzania showed that the use of the EIR decreased over time in settings where it was used in parallel with paper-based documentation compared to exclusive use [32]. In most other LMICs, paper-based documentation and reporting consume a significant proportion of health workers’ time, which can be alleviated by well-implemented digital tools co-designed with the end user [31,33].

Organizations play a key role in supporting the adoption of digital systems directly and indirectly and sometimes inadvertently skewing priorities [20,30]. For instance, in our study, health workers are provided with performance-based financing based on BCG vaccine coverage rates, which might explain the relatively better completeness of these data in the e-Tracker.

### Strengths

This study was conducted in sub-Saharan Africa, where there has been relatively limited research on EIRs and DHIs in general. Our findings are reasonably generalizable to the Rwandan context for two main reasons: (1) we sampled health centers at different stages of e-Tracker use, ranging from low to high, and (2) we included all users of the e-Tracker (immunization nurses, data managers, and supervisors).

Most studies that have applied the HOT-Fit framework have used quantitative methods to evaluate the effectiveness. We chose qualitative methods to gain an in-depth understanding of user-reported barriers and opportunities for e-Tracker use [20]. Our research is aligned with the national health system priorities to improve data use in the immunization program [34]. Key stakeholders, including representatives from the Ministry of Health and the Rwanda Biomedical Center, were involved at every stage of the research. They were consulted and presented with the study plan and results.

### Study Limitations

This study has some limitations. The study was conducted in 2021, after a relatively short period of e-Tracker use by the health centers. Since the first introduction of the tool, some improvements have been implemented and these were not captured in our study. For example, nationwide linkages between the CRVS and immunization registry have recently been established and health workers providing immunization can retrieve information about the child from the CRVS. The COVID-19 pandemic and the subsequent restrictions in the years following the implementation of the e-Tracker may have affected training, use, and perceptions. Health workers from the immunization program (immunization nurses, data managers, and EPI supervisors) contributed immensely to the COVID-19 response, which may have affected their attitudes and perceptions toward their general workload and e-Tracker use.

### Conclusions

The study findings revealed a low satisfaction level among the users of the immunization e-Tracker in Rwanda due to technical as well as implementation-related factors. Technical functionalities and implementation strategies co-designed with the user can help improve user experience and eventually maximize the benefits of the e-Tracker. Implementation strategies to reduce or remove dual documentation on paper and digital systems and to generate automated digital monthly immunization reports can save valuable time for health workers.

## Appendix

Multimedia Appendix 1 COREQ (Consolidated Criteria for Reporting Qualitative Research) checklist.

Multimedia Appendix 2 Interview guides.

## Abbreviations

BCG: Bacille Calmette-Guérin
COREQ: Consolidated Criteria for Reporting Qualitative Research
CRVS: Civil Registration and Vital Statistics
DHI: digital health intervention
EIR: electronic immunization registry
EPI: Expanded Program on Immunization
HMIS: health management information system
HOT-Fit: Human, Organization, and Technology–Fit
LMIC: low- and middle-income country
