# Supplementary material for: Electronic Immunization Registry in Rwanda: Qualitative Study of Health Worker Experiences
Source: JMIR Hum Factors. 2024 May 28;11:e53071. doi: 10.2196/53071 (PMC11177796; doi:10.2196/53071)
Supplement: Multimedia Appendix 2 [file humanfactors_v11i1e53071_app2.pdf]

Interview guides

Article title: Electronic immunization registry in Rwanda: a qualitative study of health worker experiences.

Questions for immunization nurses

1. How would you describe your current duties and roles in the immunization program?
2. How long have you been working as immunization nurse in this health center?
3. What do you like most about your service?
    a. Probe: Consultation, Vaccination, Recording and Reporting and/ or data transfer to e-Tracker.
4. What do you like least about your service?
    a. Probe: Consultation, Vaccination, Recording and Reporting and/ or data transfer to e-Tracker.
5. Whom are you working with in your service?
6. How do you describe your working environment?
    a. Probe: In terms of the physical working environment
    b. Probe: Interaction with supervisors and colleagues.
    c. Probe: How are supervision and follow up activities from district level being done at health facility by your supervisor?
7. Do you do immunization outreach activities? If yes, could do you describe how easy or difficult it is to record data during immunization outreach sessions? What are the difficulties?
    a. Optional: If answer number 7 is "YES". How do you relate /compare data recorded during outreach sessions to the data recorded on fixed site sessions? Please describe briefly.
    b. Probe: in terms of quality and completeness of data: for example, on a busy working day, are you able to complete the documentation?
8. Could you describe how easy/difficult it is to achieve the following in your catchment area?

    a. Immunization coverage
    b. Equity: are you able to vaccinate children of - poorer vs. rich, hard-to-reach areas, less educated mothers vs. more educated mothers.
    c. Probe: What creates the difficulty? Why is it difficult to achieve 100% coverage? Why is it difficult to achieve equity?
9. Did you receive training in the use of the immunization e-Tracker? Do you use/have you used the e-Tracker in your work? If yes, what do you use it for? If not, why?
10. Could you describe your current skills level of using immunization e-Tracker?
    a. Probe: Do you own a smartphone? Do you use a computer or tablet outside of your work? If yes, for what and how often – e.g. Browsing social media? Have you received in-service training in the use of digital tools?
11. How would you describe the e-Tracker?
    a. Probe: Is it easy to use? Was it easy to learn? What do you think about the system response time?
12. How do you access technical support for the e-Tracker if needed? How fast and slow is it upon submission of your request? Does it have any effect on your everyday work?
13. Has the e-Tracker changed the way you work?
    a. Probe: To what extent do you accept or resist the changes brought by the e-Tracker? Why?
    b. Probe: Do you perceive your documentation burden has become more or less with the introduction of the e-Tracker? If more, how? If less, how?
14. Does the e-Tracker fit your daily workflow? Why and why not?
    a. Probe: How can the e-Tracker better fit your workflow?
15. Are you satisfied with immunization e-Tracker functionalities? Why/why not?
    a. Probe: Is the data entry interface user-friendly? Can you navigate the system easily? Can you find individual records in the system easily?
16. Does the e-Tracker capture data that is/could be useful to you?
    - Probe: In terms of indicators and data elements/variables
    - Probe: Are there data elements that are not useful? If so, why?
17. Do you have concerns about data security in the e-Tracker?

    a. Probe: Do you have concerns on data sharing? On who uses the data, or has access to the data?

18. How do you find the quality of data in registers compared to the quality of data in the e-Tracker?
    a. Probe: Which source has more complete data? Which source has more accurate data? Which source is more reliable?
19. According to your experience, how do you describe the usefulness of immunization e-Tracker since its implementation?
    a. Probe: Which aspects/factors of registers versus e-Tracker are helpful to you today?
20. On a personal note, what are your urgent needs in terms of digital tools in immunization e-Tracker implementation that would facilitate your job as responsible nurse?
    - Features
    - Time management
    - Replication of duties (work assignments)
    - Recording
    - Reporting
    - Transfer
    - Feedback mechanisms with beneficiaries and system users (all).
21. If you were given a digital tool to use during an immunization session, how do you imagine that will work?
    - Explain with a demo/poster/video what this means.
    - Probe:
        - What would be the difficulties of such a system? What would be the advantages of such a system?
        - Clinical decision support/automated checklists
        - Automated and streamlined reporting (minimize documentation burden)
        - Automated feedback dashboards with targets
        - SMS messages/alerts to self, to mothers of children

22. Is there any other thing that we have not talked about in this conversation that you would want to talk about?

Questions for data managers

1. How would you describe your current duties and roles in the immunization program?
2. How long have you worked as a health center data manager?
3. What do you like most about your service?
    - Probe: Data entry, interaction with health workers
4. What do you like least about your service?
    - Probe: Data entry problems
5. Whom are you working with in your service?
6. How do you describe your working environment?
    - Probe: In terms of the physical working environment
    - Probe: Interaction with supervisors and colleagues.
    - Probe: How are supervision and follow-up activities from the district level being done at the health facility by your supervisor?
7. Can you describe the data management activities you do as part of your work?
    - Probe: Do you check for data discrepancy, data entry and/or do data transfer to e-Tracker?
    - Probe: Is your work environment favourable in fulfilling your duties?
8. Could you describe your current skill level in using the immunization e-Tracker?
9. Have you been trained in the immunization e-Tracker? Do you use/have you used the e-Tracker? If yes, what do you use it for? If not, why?
10. How would you describe the e-Tracker?
    - Probe: Is it easy to use? Was it easy to learn? What do you think about the system response time?
11. How do you access technical support for the e-Tracker if needed? How fast and slow is it upon submission of your request? Does it have any effect on your everyday work?
12. Do you have concerns about data security in the e-Tracker?
    - Probe: Do you have concerns about data sharing? On who uses the data, or has access to the data?

13. How do you find the quality of data in registers compared to the quality of data in the e-Tracker?
    - Probe: Which source has more complete data? Which source has more accurate data? Which source is more reliable?
14. How do you see the quality of data brought to you for entering them into the e-Tracker?
    - Probe: How complete do you find immunization data when transferring them into the e-Tracker?
    - Probe: How much of quality control and consistency checks do you have to do, before entering data into the e-Tracker?
15. On a personal note, what are your essential needs in terms of digital tools in the immunization e-Tracker implementation that would facilitate your job as data manager?
    - e-Tracker features
    - Time management
    - Duplication of efforts (work assignments)
    - Recording
    - Reporting
    - Data transfer
    - Feedback mechanisms within the same health setting and system users (all).
16. If care providers/health workers themselves did data entry during an immunization session, how do you imagine this will work?
    - Explain with a demo/poster/video what this means.
    - Probe:
        - What implications will this have on the data quality?
        - What implications will this have on your own work?
17. In your opinion, is there any other thing important that we have not talked about in this conversation that you would want to talk about?

Questions for EPI supervisors

1. How would you describe your current duties and roles in the immunization program?
2. How long have you been working as an immunization focal person in this district hospital?
3. What do you like most about your service?
    - Probe: One-on-one supervision, group interaction with health workers
4. What do you like least about your service?
    - Probe: One-on-one supervision, group interaction with health workers
5. Whom are you working with in your service?
6. How do you describe your working environment?
    - Probe: In terms of the physical working environment
    - Probe: Interaction with other supervisors, program managers, health workers
    - Probe: How are supervision and follow-up activities from the district level being done at the health facility by the supervisor?
7. Have you been trained in the immunization e-Tracker? Do you use/have you used the e-Tracker? If yes, what do you use it for? If not, why?
8. Could you describe your current skills level in using the immunization e-Tracker?
    - Probe: Do you own a smartphone? Do you use a computer or tablet outside of your work? If yes, for what and how often – e.g., Browsing social media? Have you received in-service training in the use of digital tools?
9. How would you describe the e-Tracker?
    - Probe: Is it easy to use? Was it easy to learn? What do you think about the system response time?
10. Has the e-Tracker changed the way you work?
    - Probe: To what extent do you accept or resist the changes brought by the e-Tracker? Why?
    - Probe: Do you use the e-Tracker actively for supervision? How?

11. According to your experience, how do you describe the usefulness of the immunization e-Tracker since its implementation for your day-to-day activities?
    - Probe: What data are you eligible to see in the immunization e-Tracker? How often do you view data in the e-Tracker?
    - Probe: Do you give feedback to health workers based on the data entered in the e-Tracker?
12. As a supervisor, do you do data quality reviews? If yes, during the data quality review - how could you compare the quality of data in registers during your supervision and the quality of data in e-Tracker?
    - Probe: Which source has more complete data? Which source has more accurate data? Which source is more reliable?
    - Probe: which source provides the data you need for your work?
13. Do you take any decision based on immunization data in e–Tracker regarding your zone?
    - Probe: What kind of decisions?
    - Probe: How does the data quality in the e-Tracker affect your decisions?
14. How do you imagine that will work if care providers/health workers were given a digital tool to use during an immunization session?
    - Explain with a demo/poster/video what this means.
    - Probe:
        - What would be the difficulties of such a system? What would be the advantages of such a system?
        - Clinical decision support/automated checklists
        - Automated and streamlined reporting (minimize documentation burden)
        - Automated feedback dashboards with targets
        - SMS messages/alerts to self, to mothers of children
15. On a personal note, what are your essential needs in terms of digital tools in the immunization e-Tracker implementation that would facilitate your job as focal person?
    - e-Tracker features
    - Time management
    - Duplication of efforts (work assignments)

- Recording
- Reporting
- Data transfer
- Feedback mechanisms within the same or different health setting and systems (all).

16. In your opinion, is there any other thing important that we have not talked about in this conversation that you would want to talk about?
